# The pattern of expression and prognostic value of key regulators for m^7^G RNA methylation in hepatocellular carcinoma

**DOI:** 10.3389/fgene.2022.894325

**Published:** 2022-09-02

**Authors:** Jianxing Chen, Shibin Yao, Zhijuan Sun, Yanjun Wang, Jili Yue, Yongkang Cui, Chengping Yu, Haozhi Xu, Linqiang Li

**Affiliations:** ^1^ College of Chemistry and Life Science, Chifeng University, Chifeng, China; ^2^ Department of General Surgery, The First Affiliated Hospital of Harbin Medical University, Harbin, China; ^3^ Department of Emergency, Affiliated Hospital of Chifeng University, Chifeng, China; ^4^ International Education School, Chifeng University, Chifeng, China; ^5^ Department of Pediatrics, Affiliated Hospital of Chifeng University, Chifeng, China; ^6^ Department of General Surgery, Affiliated Hospital of Chifeng University, Chifeng, China; ^7^ Key Laboratory of Hepatosplenic Surgery, Ministry of Education, Harbin Medical University, Harbin, China

**Keywords:** m7G, hepatocellular carcinoma, bioinformatics, prognosis, risk signature

## Abstract

N7-methylguanosine (m^7^G) modification on internal RNA positions plays a vital role in several biological processes. Recent research shows m^7^G modification is associated with multiple cancers. However, in hepatocellular carcinoma (HCC), its implications remain to be determined. In this place, we need to interrogate the mRNA patterns for 29 key regulators of m^7^G RNA modification and assess their prognostic value in HCC. Initial, the details from The Cancer Genome Atlas (TCGA) database concerning transcribed gene data and clinical information of HCC patients were inspected systematically. Second, according to the mRNA profiles of 29 m^7^G RNA methylation regulators, two clusters (named 1 and 2, respectively) were identified by consensus clustering. Furthermore, robust risk signature for seven m^7^G RNA modification regulators was constructed. Last, we used the Gene Expression Omnibus (GEO) dataset to validate the prognostic associations of the seven-gene risk signature. We figured out that 24/29 key regulators of m^7^G RNA modification varied remarkably in their grades of expression between the HCC and the adjacent tumor control tissues. Cluster one compared with cluster two had a substandard prognosis and was also positively correlated with T classification (T), pathological stage, and vital status (fustat) significantly. Consensus clustering results suggested the expression pattern of m^7^G RNA modification regulators was correlated with the malignancy of HCC strongly. In addition, cluster one was extensively enriched in metabolic-related pathways. Seven optimal genes (*METTL1*, *WDR4*, *NSUN2*, *EIF4E*, *EIF4E2*, *NCBP1*, and *NCBP2*) were selected to establish the risk model for HCC. Indicating by further analyses and validation, the prognostic model has fine anticipating command and this probability signature might be a self supporting presage factor for HCC. Finally, a new prognostic nomogram based on age, gender, pathological stage, histological grade, and prospects were established to forecast the prognosis of HCC patients accurately. In essence, we detected association of HCC severity and expression levels of m^7^G RNA modification regulators, and developed a risk score model for predicting prognosis of HCC patients’ progression.

## Introduction

In 2020, the sixth most commonly diagnosed cancer is primary liver cancer (∼906,000 new cases) and the third global lethal cancer (∼830,000 deaths) (Sung et al., 2021). HCC comprised 75–85% of primary liver cancer cases (Sung et al., 2021). Epidemiological studies have shown that chronic infection with hepatitis B or C viruses, ingestion of aflatoxin-contaminated foods, excessive drinking, overweight, type II diabetes, and smoking are the main threats for HCC (Thomas London et al., 2018). At early stages, most diagnosed patients of HCC are firstly identified in an advanced stage because of symptomless nature of HCC which results in limited curative options and poor prediction for one or both intrahepatic and extrahepatic metastasis (Llovet et al., 2018). For advanced-stage cases of HCC, only 1/3 patients can benefit from chemotherapy by using one or multiple kinase inhibitors, and the regimen brings evident drug resistance or toxicity with long-term therapeusis, suggesting no effective treatment can effectively improve the outcome of HCC (Wang et al., 2021). Presently, TNM staging system (Tumor T, Node N, and metastases M) remains the most extensively used predictive index to monitor HCC progression. Whereas, HCC is most diversified; thus, the same TNM stage patients tended to show significant differences in survival outcomes and responses of treatment. Hence, a finer comprehension of HCC lying molecular mechanisms can enhance dissatisfying outcomes in patients and identify new and reliable molecular signatures for predicting prognosis.

N7-methylguanosine (m^7^G) methylation is a very frequent RNA modification, which recently has shown an important role in balancing gene expression in both prokaryotes and eukaryotes (Wang et al., 2012; Létoquart et al., 2014; Haag et al., 2015). The m^7^G modification is widespread in cells for engaging in different intracellular biological function, such as transcription elongation, pre-mRNA splicing, nuclear export and mRNA translation etc. m^7^G modification has comprehensive effects on tRNA and mRNA (in the cytoplasm, an m^7^G methyltransferase complex, METLL1-WDR4, mediated m^7^G modification of tRNAs and mRNAs, regulating pre-mRNA splicing, RNA export, stability, and translation), and rRNA (in nucleus, 18S rRNA m^7^G modification mediated by WBSCR22, thus, the evolution of rRNAs and biosynthesis of 40S ribosomal subunit were regulated) molecules posttranscriptionally, and are associated with many biological processes, including the occurrence and development of human diseases (Jonkhout et al., 2017; Enroth et al., 2019; Rong et al., 2021). In mammals, deficiency in m^7^G tRNA modification is associated with developmental diseases (Shaheen et al., 2015; Braun et al., 2018). In addition, m^7^G mRNA modification participate in the pathogenesis of multiple human cancers (Rong et al., 2021). METTL1, a m^7^G methyltransferase, is upregulated in tumor-bearing patients compared to healthy controls (Liu et al., 2019; Tian et al., 2019). m^7^G exhibits the potential to become a biomarker for some types of cancer (Rong et al., 2021). These results indicate m^7^G modification plays conclusive roles in many diseases, involving tumors. However, the relationship between m^7^G modulators and tumors’ progression remains unclear, and requires a great attention and in-depth research. Therefore, further understanding of the roles of m^7^G regulators in cancer development might provide an attractive perspective for cancer therapy (Rong et al., 2021).

The cause of poor prognosis of cancer is its special invasive biological characteristics, such as epithelial-mesenchymal transition (EMT), signaling transduction, cancer stem cell formation, tumor angiogenesis and cancer metabolism, etc. (Zhang et al., 2020) Researches, recently, have revealed that abnormal m^7^G RNA modifications play crucial part in cancer progression. For example, the hepatocarcinogenesis *in vitro* and hydrodynamic transfection HCC mouse models could be promoted by METTL1-dependent m^7^G tRNA modification (Chen Z. et al., 2021). m^7^G tRNA modification and METTL1 are raised in HCC and correlated with worsening prognosis (Chen Z. et al., 2021). Overexpression of METTL1 suppresses colon cancer (CC) cells proliferation, invasion, migration, and induces cell apoptosis in a m^7^G dependent manner (Liu et al., 2020). The overexpression of METTL1 increases chemosensitivity of CC cells to cisplatin, because within the carcinoma treating process, the chemoresistance regulating process was crucially affected by METTL1-mediated m^7^G (Liu et al., 2019). *In vitro* and *in vivo*, the EGFR pathway genes and the genes involved in cell-cycle were scrupulously regulated by METTL1-mediated m^7^G tRNA modification and thus promoted intrahepatic cholangiocarcinoma (ICC) progression (Dai et al., 2021). The elevation of METTL1 and WDR4 were reported to be related with worse prognosis of human lung cancer and intrahepatic cholangiocarcinoma negatively associated with patient (Ma et al., 2021; Orellana et al., 2021). Two kinds of m^7^G tRNA modifying enzymes, METTL1 and NSUN2, determine 5-Fluorouracil (a drug used in the treatment of cancer) sensitivity in human cancer cells (Okamoto et al., 2014). In summary, the malignancy of cancer can significantly be advanced by the deregulation of m^7^G modification. This describes clearly that m^7^G RNA modification has prognostic effects in HCC patients. According to a review for m^7^G (Tomikawa, 2018), 29 related genes were downloaded from the Gene Set Enrichment Analysis (GSEA) database for subsequent studies.

In this exploration, the transcriptomic data of HCC from The Cancer Genome Atlas (TCGA) dataset is used to evaluate the expression profiles of the 29 key regulators of m^7^G RNA modification. Furthermore, based on the expression patterns of m^7^G RNA modification regulators, HCC cases were classified into 2 clusters via consensus clustering, and these 2 clusters displayed outstandingly contrasting clinical consequences. Moreover, based on risk signature, a prognostic prediction model was established, which had approving value of prediction for HCC patients. In addition, the prognostic correlation of this risk indication was successfully verified in the Gene Expression Omnibus (GEO) database. Our work helps to elucidate the important roles of m^7^G modification regulators in tumors’ progression and signifies the prospect of the genetic expression signature of m^7^G regulators in predicting the prognosis of HCC, which will contribute to the providing of personalized prognosis of clinical outcomes and pointing out a new orientation for targeting drugs discovery in HCC patients.

## Materials and methods

### Data collection

The transcriptome data of RNA-seq and the relevant clinical information of HCC patients were acquired from TCGA (website: https://portal.gdc.cancer.gov/; until 10 February 2022). For further analysis, 374 HCC tissues and 50 normal adjacent tumor tissues were involved altogether.

Currently, 29 known genes, including METTL1, WDR4, NSUN2, DCP2, DCPS, NUDT10, NUDT11, NUDT16, NUDT3, NUDT4, NUDT4B, AGO2, CYFIP1, EIF4E, EIF4E1B, EIF4E2, EIF4E3, GEMIN5, LARP1, NCBP1, NCBP2, NCBP3, EIF3D, EIF4A1, EIF4G3, IFIT5, LSM1, NCBP2L, and SNUPN are recognized as the regulators of m ^7^G methylation. For subsequent analyses, the transcribed profiles of the 29 key regulators were recovered from the HCC cohort of the TCGA database. An irrelevant cohort (GSE54236) derived from the GEO (http://www.ncbi.nlm.nih.gov/geo) database and containing 78 HCC cases with relevant mRNA expression data and the survival information was used for external verification.

### Bioinformatics analysis

Between the RNA composition of HCC samples and normal tissues, we screened the differentially expressed genes (DEGs) which encoded the m^7^G RNA methylation regulators at different levels via the use of the Wilcoxon test method in R software (version 4.1.0). False discovery rate (FDR), a more liberal criterion, was adopted together with absolute log2 fold change (FC) to determine the significance criteria (FDR <0.05, and FC > 1). Thereafter, we used a vioplot to show the 29 m^7^G-associated genes’ expression in 374 HCC and 50 control tissues. In order to survey the correlation between any two regulators of m^7^G RNA methylation, the Spearman correlation analyses were performed via R software.

To evaluate the association between the expression of m^7^G RNA methylation regulators and HCC prognosis, we used the “ConsensusClusterPlus” R package to cluster the HCC cohort into two distinct subgroups. Then, the principal component analysis (PCA) was performed via the “limma” and “ggplot2” packages to confirm the classification outcomes. A survival curve was constructed to contrast survival between clusters on the basis of the Kaplan-Meier analysis log-rank test. We applied the Chi-square test to determine the differences in clinical parameters between the 2 clusters. To functionally annotate the genes differentially expressed in the two subgroups, the Gene Ontology (GO) and the Kyoto Encyclopedia of Genes and Genomes (KEGG) analyses were performed.

We used Univariate Cox regression analyses to evaluate the relationship between m^7^G-associated genes’ expression and overall survival (OS). Afterward, to keep away overflow, the least absolute shrinkage and selection operator (LASSO) Cox regression was performed to remove the high relevant genes by using the “glment” package. Ultimately, a risk signature of a seven-m^7^G-regulatory-gene was determined. In order to yield risk scores, we multiplied the gene expression and its coefficient acquired from the LASSO Cox regression. HCC cases were then allocated to the low-risk and high-risk groups by using median risk scores. By employing the “survival” package, the Kaplan-Meier analysis was executed to generate and analyze survival-time data. Receiver operating characteristic (ROC) curves were applied to inspect the prognostic accuracy of the predicted results derived from the model. The differences of clinicopathological variables between the low- and the high-risk groups were visualized by a heatmap and examined by Chi-square test. Moreover, as effective means, univariate and multivariate Cox regression analyses were performed to assess whether the risk scores were independent and appropriate prognostic indicators. To verify the prognostic value of th e seven-m^7^G-regulatory-gene risk signature, the GSE54236 dataset was applied as a validated cohort (Villa et al., 2016). Risk score of each patient was computed based on the formula described earlier. By applying the same cutoff criteria, all HCC patients were classified into low- and high-risk groups. Thereafter, the Kaplan-Meier analysis and the ROC curve analysis were respectively executed for evaluating the prognostic values.

Ultimately, a prognostic nomogram was established using clinical information (age, gender, histologic grade, and pathological stage) and risk scores to forecast 1-, 3-, and 5-years survival in HCC patients by the “rms” package.

From http://www.bioconductor.org, we acquired all of the aforementioned R packages.

The pipeline of the research was shown in Supplementary Figure S1.

### Statistical analysis

For all statistical analyses, R software (version 4.1.0) was utilized. The significance of the threshold was *p* < 0.05.

## Results

### Recognition of m^7^G RNA modification regulators expressed differentially in HCC

**FIGURE 1:**
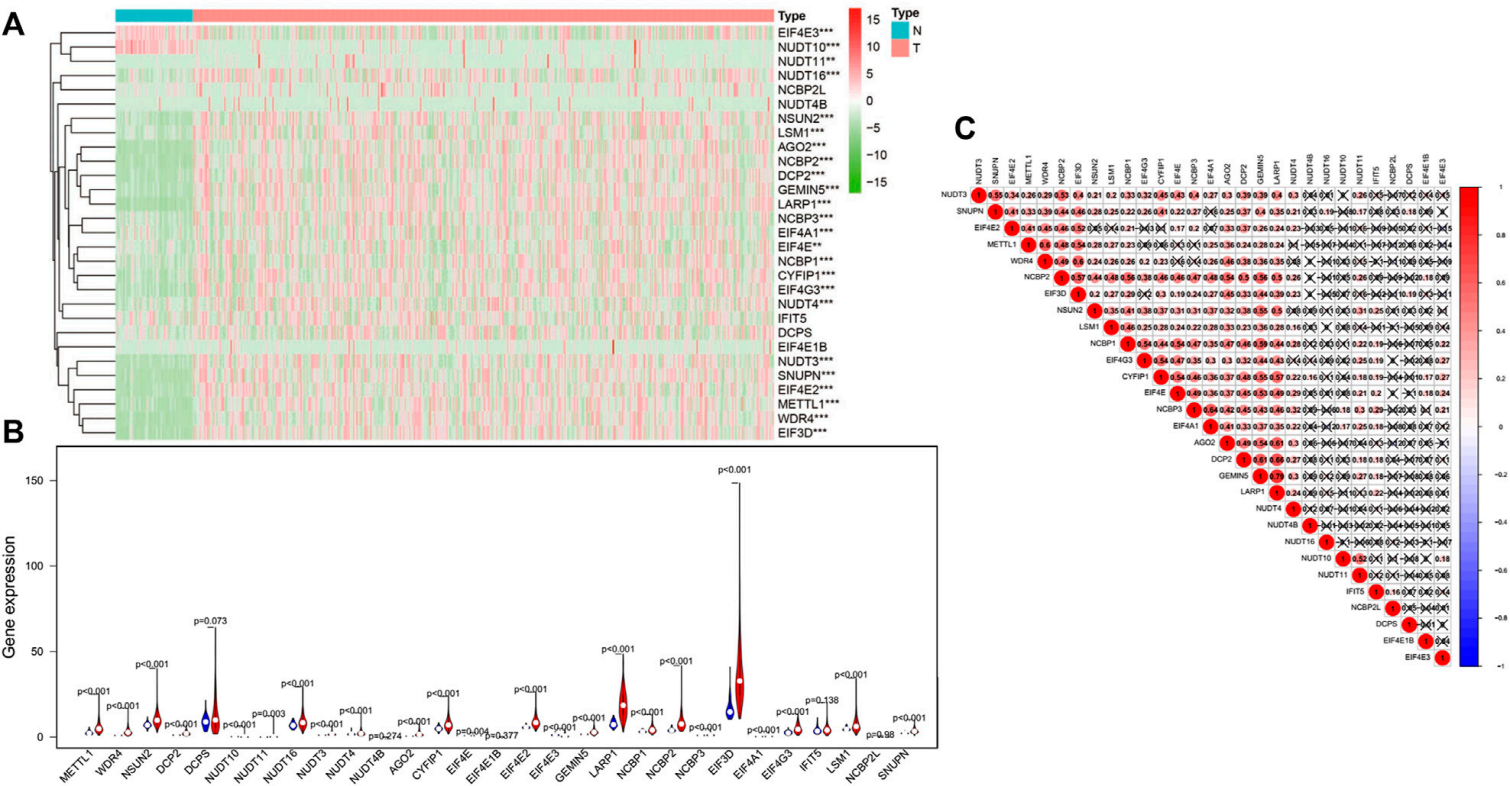
The levels of m^7^G modification regulators in hepatocellular carcinoma (HCC). **(A)**The heatmap shows the mRNA level of m^7^G RNA modification regulators in each sample. “N” represents normal sample, and “T” represents cancer sample. Green stands for low expressions, and red stands for high expressions. **(B)** The vioplot shows the differential regulators of m^7^G RNA modification in HCC. Blue stands for normal samples, and red stands for HCC samples. White spots indicate the median value of the expression. **(C)** Spearman correlation analyses of 29 m^7^G RNA modification regulators in HCC. ***p < 0.001.

An analysis of 29 differential expressed m^7^G regulatory genes was performed in HCC (*n* = 374) and normal tissues (*n* = 50). The majority of m^7^G-associated genes (24/29) were expressed between HCC and adjacent tissues differentially showed by heatmap clearly (Figure 1A). Particularly, the levels of expressions for NUDT16, NSUN2, LSM1, AGO2, NCBP2, DCP2, GEMIN5, LARP1, NCBP3, EIF4A1, NCBP1, CYFIP1, EIF4G3, NUDT3, SNUPN, EIF4E2, METTL1, WDR4, and EIF3D in cancer tissues were significantly higher (all *p* < 0.001) than those in normal tissues. However, the higher expression levels of EIF4E3 and NUDT10 (both *p* < 0.001) were remarkably in normal tissues than those in tumor samples. No significant difference was found in DCPS, NUDT4B, EIF4E1B, IFIT5 and NCBP2L (all *p* > 0.05) (Figure 1B). Furthermore, we performed an association analysis to characterize further inherent connection among 29 m^7^G RNA modification regulators. As indicated in Figure 1C, the heatmap analysis showed that the connection between GEMIN5 and LARP1 was the most remarkable (r = 0.79). The most likely positive correlated expression level of GEMIN5 was with LARP1.

### Identify two clusters of HCC patients with different clinical outcomes by using consensus clustering based on m^7^G RNA modification regulators

**FIGURE 2:**
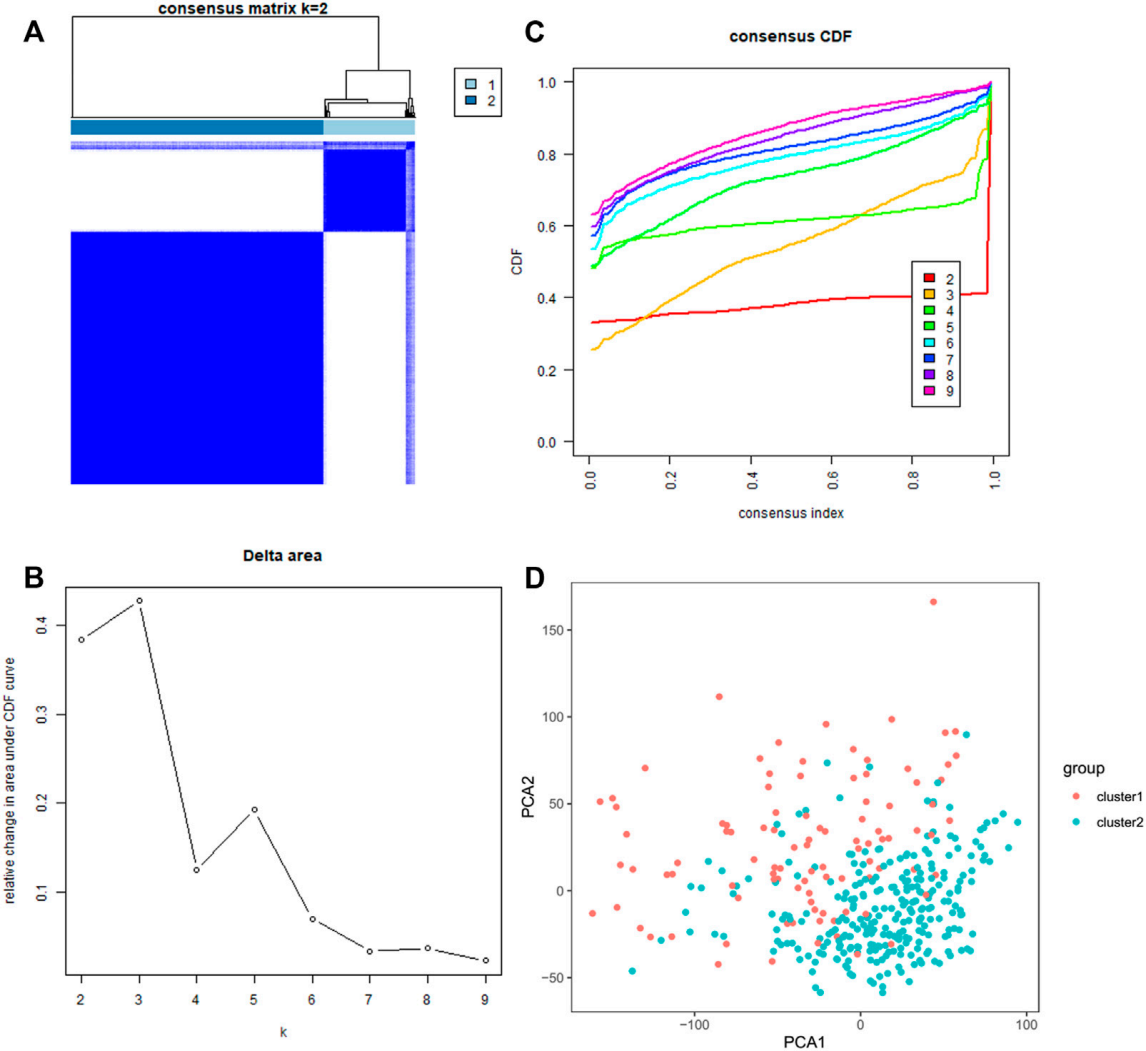
Consistent clustering analyses of the HCC. **(A)** The correlations between subgroups when the number of clusters is *k* = 2. **(B)** Cumulative distribution function (CDF) for *k* = 2–9 is displayed. **(C)** The relative variation of the area under the CDF curve of *k* = 2–9. **(D)** Principal component analysis of the RNA-seq data. Red dots stand for cluster 1, and cyan dots stand for cluster 2.

**FIGURE 3:**
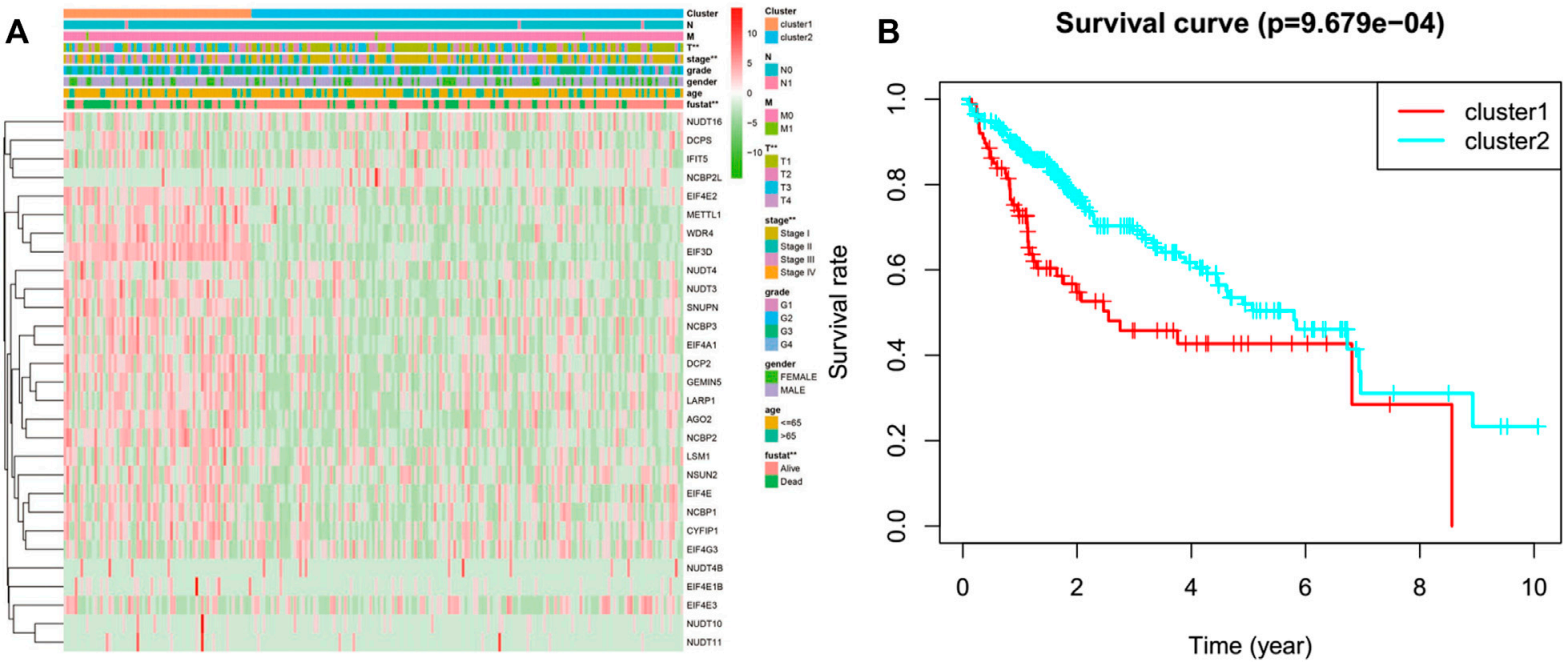
Differences between cluster one and cluster two in clinicopathological features and overall survival. **(A)** Heatmap and clinicopathological features of the two clusters. Green stands for low expressions, and red stands for high expressions. **(B)** Compare the overall survival (OS) distribution of cluster one and cluster 2. *p < 0.05, ***p < 0.001.

We classified the patients into clusters based on the patterns of mRNA expression to explore additionally the HCC clinical importance of 29 m^7^G RNA modification regulators. *k* = 2 grant the best clustering as well as the HCC cohorts could be divided into two definite and nonoverlapping clusters according to the analogy of the m^7^G RNA modification regulators (Figures 2A–C). We further performed the clustering analysis using the principal component analysis (PCA) to corroborate clustered results. As can be seen from Figure 2D, the PCA plot unveiled distinct differences between two clusters. Afterward, significant differences between the two clusters in the OS and the clinical parameters were evaluated. Hence, the OS of cluster two was more preferable than that of cluster one in significant level (*p* < 0.01) (Figure 3B). In addition, most m^7^G RNA modification regulators in cluster one exhibited higher expression levels than that in cluster 2 (Figure 3A). In contrast to cluster 2, cluster one was associated with T classification (T), later pathological stage, and vital status (fustat) significantly (all *p* < 0.001). There were no significant differences in the degree of Nodal involvement, Metastasis, tumor grading, age, and gender (Figure 3A). Therefore, the final outcomes of consensus clustering proposed that the mRNA profile pattern of m^7^G RNA modification regulators is strongly related to the malignancy of HCC.

**FIGURE 4:**
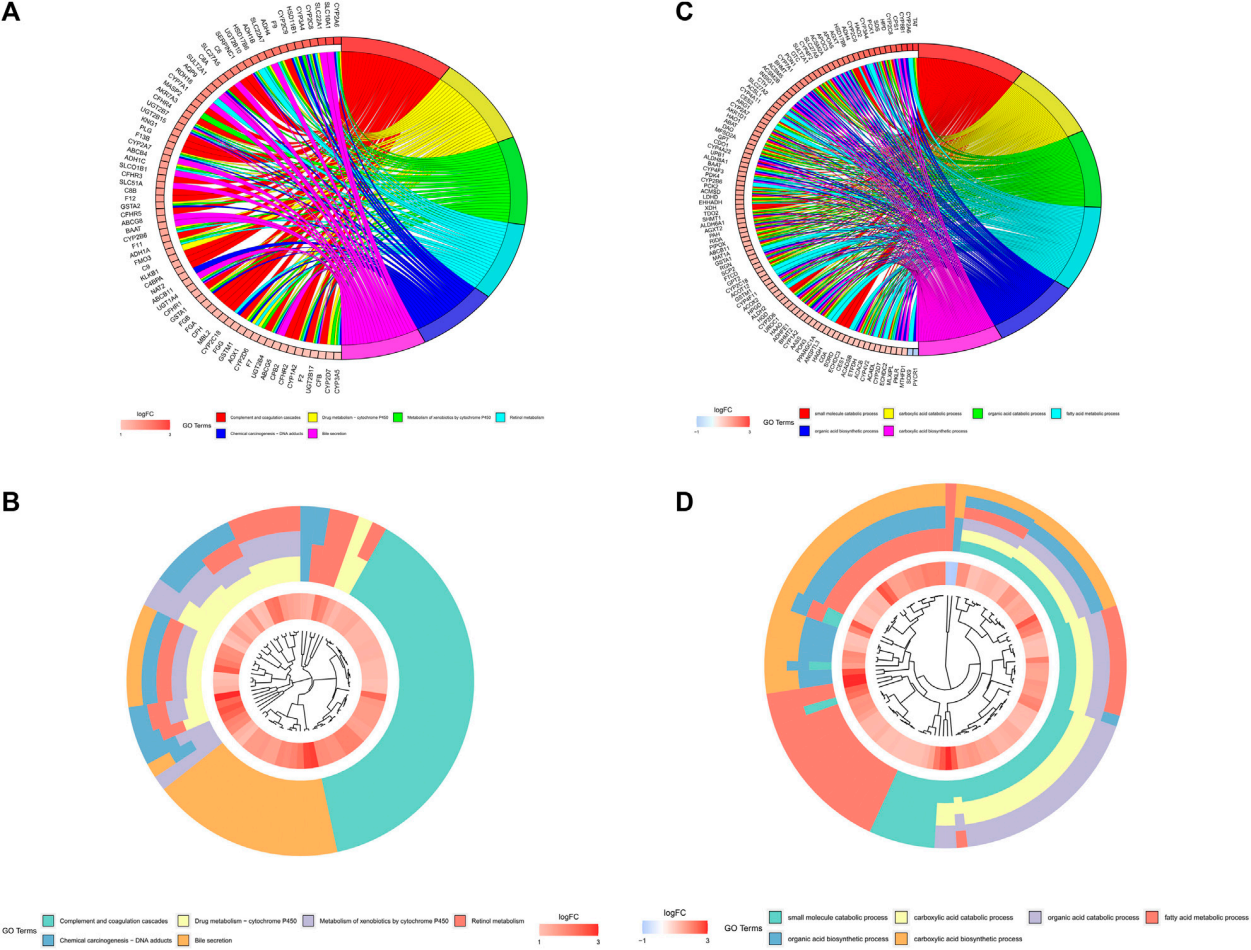
Analyses of the differentially expressed genes between the two clusters by the Kyoto Encyclopedia of Genes and Genomes (KEEG) and Gene Ontology (GO). The highly expressed genes in cluster one were functionally annotated applying the GO terms **(A,B)** and KEGG pathway **(C,D)**.

On the basis of biological processes, GO and KEGG analyses of the DEGs between two clusters were performed to explicate the clustered results. The outcomes of the GO analyses proposed that the up-regulated genes were enhancing metabolic-related biological processes, such as “small molecule catabolic process”, “carboxylic acid catabolic process”, “fatty acid metabolic process”, “organic acid catabolic process”, “organic acid biosynthetic process” and “carboxylic acid biosynthetic process” (Figures 4A,B). The KEGG analyses revealed that these up-regulated genes were adding to “Complement and coagulation cascades”, “Drug metabolism - cytochrome P450”, “Metabolism of xenobiotics by cytochrome P450”, “Retinol metabolism”, “Chemical carcinogenesis - DNA adducts” and “Bile secretion” pathways (Figures 4C,D).

### A prognostic risk model established on the basis of the mRNA level of m^7^G regulator genes

**FIGURE 5:**
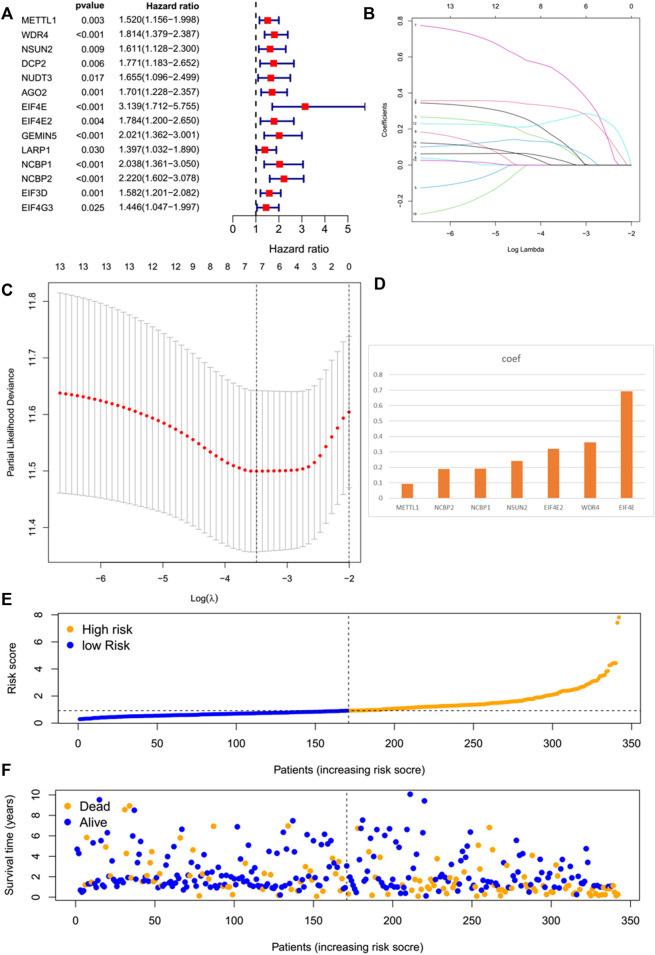
The prognostic risk model established on basis of m^7^G RNA modification regulator genes. **(A)** Univariate Cox regression analyses of m^7^G RNA methylation regulators. **(B–D)** The process of constructing signatures applying least absolute shrinkage and selection operator (LASSO) Cox regression. **(E)** Distribution of risk scores of the patients in the prognostic model. **(F)** Distributions of survival status of the patients in the prognostic model.

**FIGURE 6:**
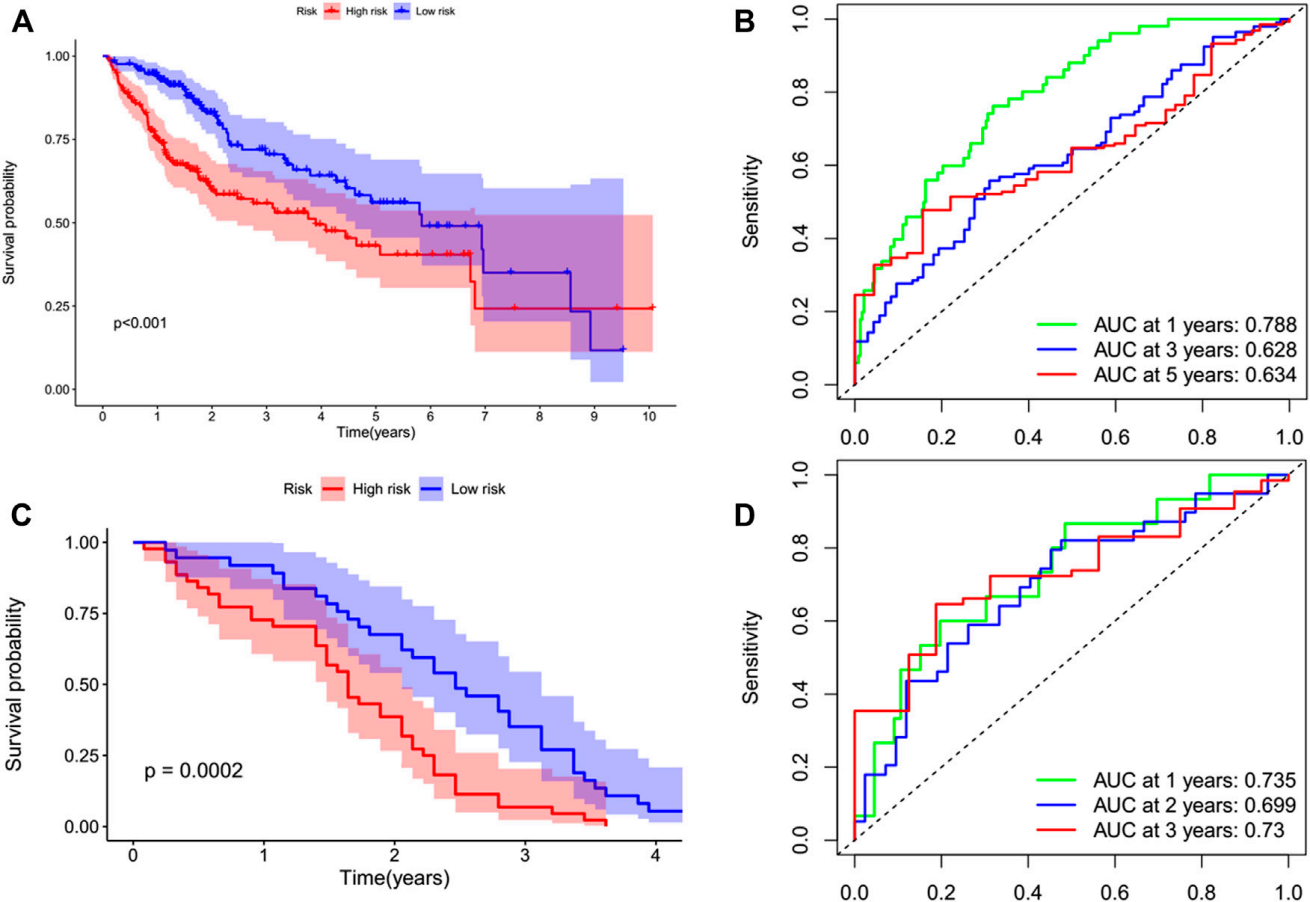
Kaplan–Meier survival analysis of the prognostic model. Invalids in both datasets were divided into high-risk (red) and low-risk (blue) groups, applying the median risk score as the threshold. **(A,B)** In the TCGA cohort, the survival probability of the low-risk group was higher than that of the high-risk group (*p* < 0.001). The AUCs at 1-, 3-, and 5-years were 0.788, 0.628, and 0.634, respectively. **(C,D)** This prognostic model was verified in the GEO cohort. The survival probability of low-risk group was higher than that of high-risk group (*p* = 0.0002). The AUCs at 1 year, 2 years, and 3 years were 0.735, 0.699, and 0.73, respectively.

**FIGURE 7:**
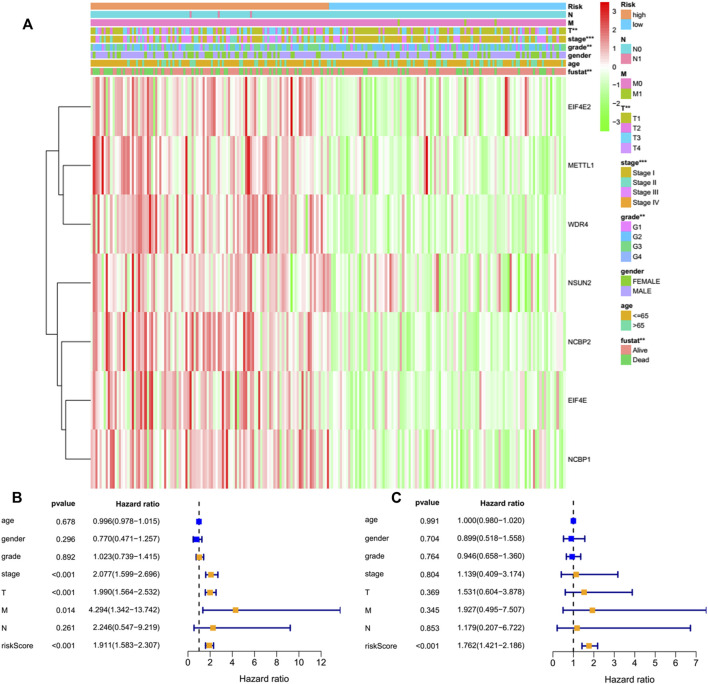
Effects of the risk score and the clinicopathological characteristics on the prognosis of invalids with HCC. **(A)** Heatmap manifests the distribution of clinicopathological characteristics and the expressions of seven m7G RNA modification regulators in high- and low-risk groups. **(B)** Univariate Cox regression analyses of the clinicopathological parameters and OS. **(C)** Multivariate Cox regression analyses of the clinicopathological parameters and OS. **p < 0.01, ***p < 0.001.

By performing a univariate Cox regression analysis on the mRNA levels of 29 key regulators, we specified a secure correlation between m^7^G RNA methylation regulators and the prognosis in HCC invalids. The results displayed those 14/29 regulators were outstandingly associated with OS (*p* < 0.05) (Figure 5A). The 14 regulators are all considered as risky genes with HR > 1. Thereafter, LASSO Cox regression analysis was applied to recognize the m^7^G RNA modification regulators possessing the best prognostic power (Figures 5B,C). Finally, seven effective genes (METTL1, WDR4, NSUN2, EIF4E, EIF4E2, NCBP1, and NCBP2) were selected to establish the risk model for HCC and also the corresponding coefficients calculated from the LASSO algorithm (Figure 5D). The risk score was calculated thus: RISK SCORE = (0.092 ∗ EXP value of METTL1) + (0.189 ∗ EXP value of NCBP2) + (0.193 ∗ EXP value of NCBP1) + (0.242 ∗ EXP value of NSUN2) + (0.320 ∗ EXP value of EIF4E2) + (0.362 ∗ EXP value of WDR4) + (0.693 ∗ EXP value of EIF4E). A ccording to the median risk score, HCC invalids were grouped as low-risk and high-risk to survey the prognostic function of the seven-gene signature model. Survival analysis showed that OS was worse in invalids with high-risk scores compared with low-risk scores (Figure 6A, *p* < 0.001). The 5-years OS rates of high-risk and low-risk group were 44.6% and 55.4%, respectively. Furthermore, ROC curve analyses were executed to estimate the area under the curve (AUC) of 0.788, 0.628, and 0.634 for the 1-, 3-, and 5-years OS, respectively. This showed eminent prognosticative power for survival results (Figure 6B). Besides, the distribution of risk scores for HCC invalids were also constructed (Figure 5E). For manifesting the survival status of each invalid, a dot pot was plotted (Figure 5F). The heatmap showed the mRNA levels of seven prognosticative genes in the groups of high-risk and low-risk (Figure 7A). Simultaneously, correlated clinical information was plotted above the heatmap. While contrasting the clinical parameters of the low-risk and high-risk groups, terms of T, stage, grade and vital status (fustat) were all significant at the 0.01 level.

### Verifying the prognostic signatures employing GEO database

The prognostic value of the signature of seven genes for the survival predictions can be assessed by validating them using the GEO microarray data (GSE54236) (Villa et al., 2016). In the GSE54236 cohort, a total of 78 patients with HCC were grouped in high- (*n* = 42) and low-risk (*n* = 36) based on the cutoff values of the TCGA cohort. Similarly, it was also showed by the survival analysis that the OS of HCC invalids in the low-risk group was significantly finer than in high-risk invalids (Figure 6C, *p* = 0.0002). The AUCs for the OS of 1-, 2-, and 3-years were 0.735, 0.699, and 0.73, respectively, stating that this prognostic model was able to better predict OS in HCC invalids clearly (Figure 6D). As no patients survived for more than 5 years, therefore, no 5-years ROC curve was constructed.

### The seven-gene risk signature independently forecasts the prognosis of HCC invalids

Only 226 patients were acceptable to perform the Cox regression analysis after removing deficient clinical cases. Univariate analysis disclosed that the seven-gene risk score, T, and stage were notably correlated with the OS of HCC cases (Figure 7B, all *p* < 0.001). We also performed a multivariate Cox regression analysis, revealing that the risk score was independently linked with OS in HCC invalids (Figure 7C, *p* < 0.001). All such outcomes manifested the independent prognosis of the risk signature of the seven genes for gender, age, histological grade, and pathological stage. Thus, indicating that the risk signature of these seven genes was able to serve as an independent prognosis for the patients of HCC.

### Prognostic nomogram established for HCC

**FIGURE 8:**
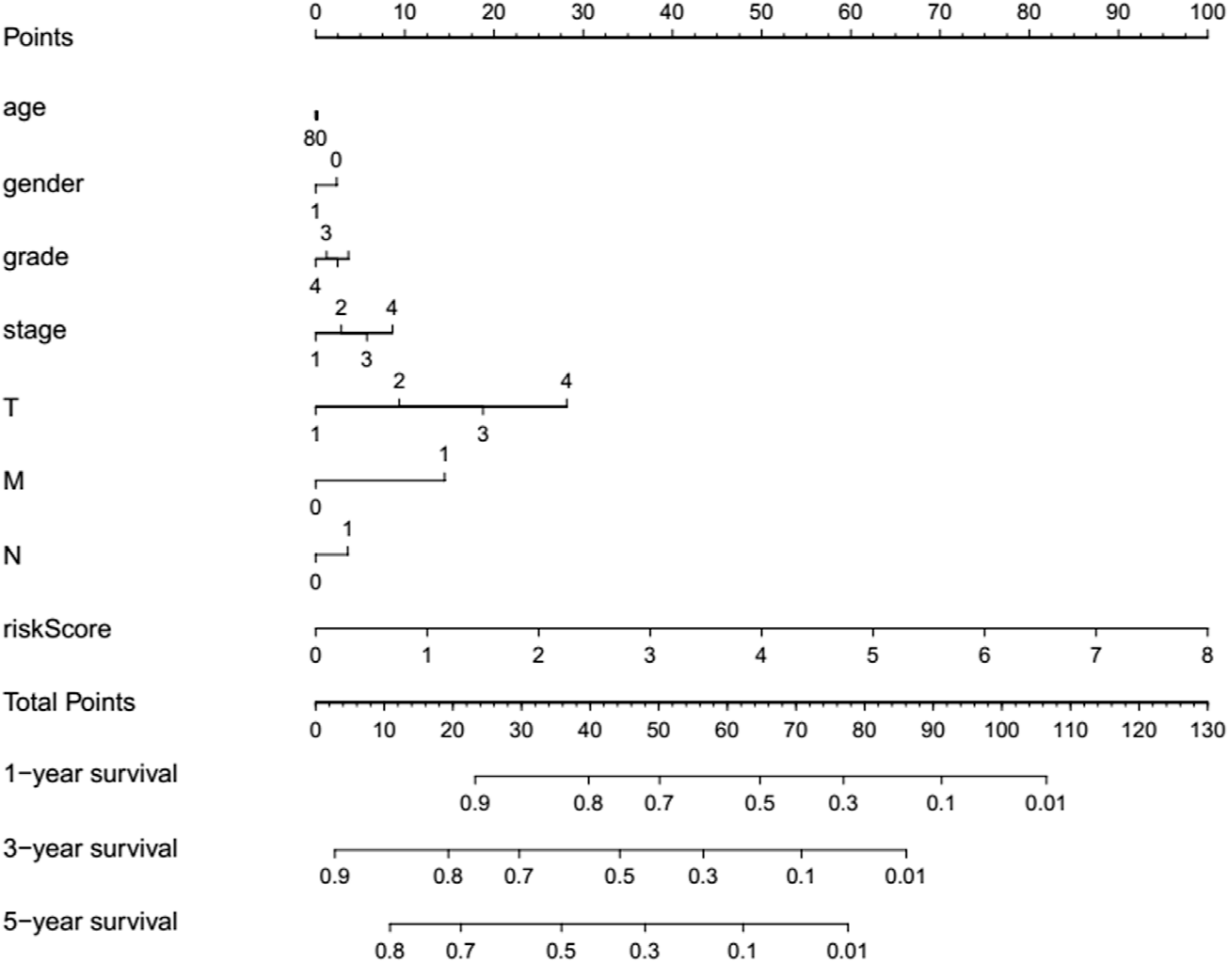
Prognostic nomogram established via the combination with the clinicopathologic features and risk score.

For providing a quantitative method to estimate the survival rate, a refreshing prognostic nomogram (Figure 8) based on age, gender, risk score, histological grade, and pathological stage was established. The results stated clearly the nomogram was able to systematically forecast the 1, 3, and 5-years OS in HCC invalids.

## Discussion

Globally, HCC is one of the most common cancers. Although great progress has been made in the diagnosis, treatment, and prognosis of HCC, which still retains the third place in cancer mortality (Sung et al., 2021). At present, there are no productive diagnostic as well as therapeutic targets for HCC. There is unsatisfactory overall survival and challenging treatment such as difficulty in predicting the prognosis, difficulties in clinical decision-making, and clinical management of patients with HCC. Hence, the molecular mechanisms implicit in contributing to tumorigenesis of HCC are very crucial and necessary to clarify. Over the years, RNA m^7^G modification has attracted great interest as a novel dimensionality of transcription regulation. Nevertheless, the research of m^7^G modification in the tumor area is yet in its infancy. As a result of the extensive appliance of RNA-seq and microarray technologies, risk scoring system in the light of multiple gene signatures is progressively used to forecast prognosis for cancers oftentimes (Chen et al., 2007; Zhang et al., 2013; Wang et al., 2019). In current study, an effective prognostic signature model through the use of seven m^7^G RNA modification regulators was established. It is encouraging that the risk score calculated from the seven-gene model could forecast the prognostic value of HCC invalids independently. Consequently, the risk signature reported in this paper can help clinicians make precise personalized survival prediction.

The prognostic model indicated all the seven m^7^G-related genes (METTL1, NCBP2, NCBP1, NSUN2, EIF4E2, WDR4, and EIF4E) might exert a stimulative effect on HCC. Tian et al. reported that METTL1 is upregulated in HCC and overexpressed METTL1 is linked to a poor prognosis of HCC (Tian et al., 2019). Overexpressed METTL1 induced oncogenic cell transformation and cancer (Orellana et al., 2021). METTL1-dependent m^7^G tRNA modification could promote hepatocarcinogenesis *in vitro* and hydrodynamic transfected in HCC mouse model, and METTL1 is raised in HCC and linked with worse prognosis (Chen Z. et al., 2021). METTL1-mediated m^7^G tRNA modification advances the progression of ICC (Dai et al., 2021). In lung cancer cases, METTL1 and WDR4 were both remarkably raised and had a negative correlation with invalids’ prognosis (Ma et al., 2021). The overexpression of METTL1 increases the chemotherapy sensitivity of CC cells to cisplatin, which is a common chemotherapy drug (Liu et al., 2019). METTL1 and NSUN2, determine 5-Fluorouracil sensitivity in human cancer cells (Okamoto et al., 2014). In function, METTL1 has not been shown to be associated with tumorigenesis, but it is in overexpression repeatedly (Orellana et al., 2021). In contrast, METTL1 is considered a latent tumor suppressor of colon cancer (Liu et al., 2020). Usually, METTL1 is considered as an oncogene. It plays an important role in cancerous cells’ growth and proliferation, invasion, and malignant phenotype transformation. Promisingly, METTL1 is a biomarker for the diagnosis and prognosis of HCC (Zhu L. R. et al., 2021). Despite being one of the most indispensable m^7^G-related genes, the overall relevance of METTL1 in cancer remains mostly unclear.

NCBP1 and NCBP2, both are the components of the nuclear cap-binding protein complex, and are essential for the processing and subcellular localization of capped RNAs. Silencing of NCBP1 reportedly results in reduced cell growth in HeLa cells (Gebhardt et al., 2015). A significant overexpression of NCBP1 was discovered in lung cancer tissues and NCBP1 promoted cancerous cells’ growth, wound healing capacity, migration, and epithelial-mesenchymal transition (Zhang et al., 2019). In the report of Zhu et al. (Zhu W. et al., 2021), an mRNA signature for prognosis prediction of liver cancer including seven mRNA was demonstrated, and most of all, like our model, NCBP2 was also included. Moreover, in their study (Zhu W. et al., 2021), the mRNA of NCBP2 in liver cancer tissues was also in significant upregulation (*p* < 0.0001) while in the comparison to normal tissues. In a meta-analysis of the molecular mechanisms of miR-193a-5p, it was found that NCBP2 was one key miR-193a-5p target gene, both of its mRNA and protein expression were significantly upregulated in lung cancer (Xie et al., 2018). In screening out the biomarkers related to chemoresistance of ovarian carcinomas, NCBP2 was selected as a potential key gene through the protein-protein interaction network analysis (Wei et al., 2015). For NCBP2 is one of the genes that is closely related to the prognosis of acute myeloid leukemia (AML), the risk score and nomogram results on pediatric AML research suggested that NCBP2 may be a risk factor for the malignant tumor (Zhang et al., 2022). These studies suggest that NCBP1 and NCBP2 are oncogenic proteins that significantly overexpressed or upregulated in cancerous tissues, as the same with our research, both of them were screened out as key genes.

WDR4, a member of the WD-repeat protein family, had been reported to play an important role in various kinds of malignant tumors (Zeng et al., 2021). In various malignant cancers, abnormal expression of WDR4 had been observed and was significantly implicated on overall survival outcomes. It is also strongly associated with tumor immunity. Furthermore, expression level of WDR4 could also be applied as one of the prognostic biomarkers of value for certain kinds of tumors (Zeng et al., 2021). Research showed the elevated WRD4 levels in cancer of liver could enhance m^7^G methylation levels remarkably and was also linked with the worse prognosis of HCC invalids (Xia et al., 2021). WDR4 is a necessary cofactor of METTL1, and coordinates with METTL1 transferring methyl groups to nucleic acids, proteins, lipids, etc., by forming a heterodimeric complex (Campeanu et al., 2021), also catalyzes m^7^G modification in tRNAs or rRNAs in eukaryotes (Jonkhout et al., 2017; Enroth et al., 2019; Rong et al., 2021). Reasonably, in our prognostic model, both METTL1 and WDR4 are included in seven key m^7^G-related genes. Mechanistically, WDR4 was identified as one kind of oncogenic protein, which regulates promyelocytic leukemia through ubiquitination negatively and promotes pulmonary tumor advance via cultivating a metastatic and immunological suppressive neoplastic microenvironment (Wang et al., 2017). The above studies testified that in the advancement of cancers, WDR4 acts as an oncoprotein, and can also be deemed as a treated target in prospect for live cancer. Therefore, as an important RNA m^7^G methyltransferase, WDR4 should be selected as a key gene in our prognostic model.

NSUN2 is not only the member of the m^7^G RNA modification system but also the m^5^C RNA member of an RNA methyltransferase, that has been proved to participate in tumorigenesis and progression of multiple cancers in an m^5^C-dependent manner (Chellamuthu and Gray, 2020; Hu et al., 2021). It has been revealed that NSUN2 is significantly involved in multiple biological functions, including cellular differentiation (Sajini et al., 2019), cellular proliferation (Xing et al., 2015), and cellular migration (Sun et al., 2019). It has been confirmed that the expression of NSUN2 in cancerous cells is higher than that in normal cells. Besides, NSUN2 promoted the proliferation and metastasis of tumor cells and the progression of gastric cancer (Hu et al., 2021) and breast cancer (Yi et al., 2017). Recently, Yan et al. reported that NSUN2 is an important intermediate element that was recruited to FOXC2 mRNA by FOXC2-AS1 (one kind of long noncoding RNA) to increase the m^5^C level of the mRNA and repress its degradation, thereby, elevating the expression of FOXC2-AS1 and advance TNM stage and shorten overall survival in gastric cancer patients (Yan et al., 2021). These studies showed that as one kind of RNA methyltransferase, NSUN2 could function through a variety of signaling pathways. However, the functional roles, overexpression mechanisms of NSUN2 and its relation between clinicopathological characteristics of tumor are still obscure. More importantly, the detailed biological functions and regulatory mechanisms of NSUN2 as a member of the m^7^G RNA modification system are still unclear.

EIF4E contributes to translation initiation, thus is the part to the eukaryotic translation initiation factor 4F (EIF4F) complex. Overexpression of eIF4E in invalids with liver cancer is associated with worse prognosis and high risk of recurrence (Jiang et al., 2016). Yang et al. reported it is through decreasing expression of EIF4E in HCC cells, that the up regulation of miR-503 prevented the proliferation of live cancer cells and increased chemosensitivity to a certain degree (Yang et al., 2017). Similarly, Zhang et al. reported it is via targeting EIF4E in HCC cells, that the inhibited miR-15a-5p promoted chemoresistance to pirarubicin (Zhang et al., 2021). Hence, targeting eIF4E may be an alternative treatment strategy for live cancer (Tan et al., 2018). It was shown that eIF4E level in colorectal cancer cell lines was higher than that in controls. Studies have also shown that high expression of eIF4E in invalids with colorectal cancer predict high risk of hepatic metastases and their related mechanism may be partly by the regulation of the levels of VEGF, cyclin D1, MMP-2, and MMP-9 (Xu et al., 2016). Li et al. discovered that the eIF4E levels in BRCA were higher than in controls, and invalids having elevated eIF4E level had lower survival and upper additive recurrence, hence they proved eIF4E level had prognosis significance for BRCA invalids (Li et al., 2021). In short, as an oncogene of several tumors, eIF4E can promote the transformation, carcinogenesis, metastasis, and chemoresistance (Pelletier et al., 2015).

EIF4E2 is an important paralog of EIF4E, suggesting that EIF4E2 acts by competing with EIF4E and block assembly of EIF4F at the cap, therefore, EIF4E2 was first characterized as a translation inhibitor. However, the pathway in which EIF4E2 participated was sensitized via hypoxia, which is a microenvironmental feature existing in numerous solid carcinomas (Uniacke et al., 2012). Furthermore, EIF4E2 is required for tumor progression, such as tumor growth in mouse xenografts (Uniacke et al., 2014). Among a variety of solid carcinomas, EIF4E2 is considered to be a valuable biomarker for distinguishing metastatic cancer from primary tumors (Ramaswamy et al., 2003). In the paper of Melanson et al., they considered eIF4E2 is like some drugs that prohibit standard translation thus, eIF4E might have the therapeutic potential in the treatment of hypoxic solid tumors (Melanson et al., 2017). Kelly et al. found it is via regulating EIF4E2 that the elevated cadherin-22 promotes the migration and invasion of cancerous cells (Kelly et al., 2018). To deepen our understanding of cancer progression, Evagelou et al. supported DDX28 as a tumor suppressor and prognostic marker through mechanistic insights into a negatively regulated hypoxic translation mediated by HIF-2α and eIF4E2 (Evagelou et al., 2020). The findings of Yang et al. showed the elevated EIF4E2 level was a distinct risk factor for prognosis of invalids with uveal melanoma, where EIF4E2 played a significant role in hypoxia related signaling pathway in the advancement of uveal melanoma (Yang et al., 2021). It was demonstrated that the phenotypic expressions of cancer genomes need to be translated through the hypoxia protein synthesis mechanism guided by eIF4E2 (Uniacke et al., 2014). These studies suggest that eIF4E2 can be related to the advancement of carcinoma and that may be an oncogenic protein that significantly overexpressed or upregulated in cancerous tissues, as the same with our research, was screened out as a key biomarker.

The results of this paper may suggest the dysregulation of m^7^G may play a significant role in the development of HCC. In the present study, heatmap (Figure 1A) exposed that many of such m^7^G associated genes (24/29) had differential expressions between HCC and controls. It should be noted that 22 of 29 m^7^G RNA methylation regulators were up-regulated in cancer tissues. On the contrary, only the levels of EIF4E3 and NUDT10 were down-regulated in HCC tissues. Osborne et al. demonstrated that EIF4E3 relies on the atypical mode of m^7^G cap recognition to act as a tumor suppressor (Osborne et al., 2013). The reduction of eIF4E3 in tumors of elevated eIF4E levels proposed that eIF4E3 identifies a related repressive mechanism in clinical disappearing into a few malignant tumors (Osborne et al., 2013). In addition, eIF4E3 level was significantly decreased in AML samples, while it was missing in HNSCC. Nevertheless, eIF4E1 expressions were considered to be extremely promoted in the two cases (Volpon et al., 2013). Considering the latent roles of eIF4E3 in inhibiting eIF4E1 functions, these carcinomas seem to be motivated through the increase of eIF4E1 carcinogenic activities and the deletion of eIF4E3 inhibitory activities (Volpon et al., 2013). Research by Kamdar *et al.* has shown that reduced level of NUDT10 may improve promoter methylation in prostate cancer by showing tumoral suppressive characteristics (Kamdar et al., 2019). Similar to our study, a recent study has been concluded that the expressions of NUDT10 mRNA and protein in gastric cancer samples were remarkably reduced than those in controls (Chen D. et al., 2021).

However, we even so admit that some restrictions of this research are worth mentioning. Initially, our data being obtained from TCGA and GEO data banks, and experimental evidences are also needed for the verification of all findings. Secondly, due to the deficiency of detected proteomic information, the differences of the protein expressions of m^7^G regulators had not been brought into bioinformatical analyses. Thirdly, the sample size differed distinctively between the cancer group and the control group, which affected the dependability of the outcomes. Fourth, certain clinical parameters related to HCC, such as alcohol consumption and, hepatitis virus DNA levels, were not considered. Ultimately, selection bias inevitably occurred, since the main patients studied are Americans and Italians. Consequently, our findings may not apply to every population.

In conclusion, we signified the prospect of the genetic expression signature of m^7^G modification regulators in predicting the prognosis of HCC. Our work has provided additional evidences for more investigation of m^7^G RNA modification in HCC. Meanwhile, METTL1, NCBP2, NCBP1, NSUN2, EIF4E2, WDR4, and EIF4E might be potential predictors and might have a prognostic value for HCC. What we found can provide personalized prognosis of clinical outcomes and point out a new orientation for targeting drugs discovery in HCC invalids. However, additional biochemical researches and functional experiments will be necessary to demonstrate our findings in the future.

## Data Availability

The original contributions presented in the study are included in the article/Supplementary Material, further inquiries can be directed to the corresponding author.
