# Supplementary figures and images for: The pattern of expression and prognostic value of key regulators for m7G RNA methylation in hepatocellular carcinoma
Source: Front Genet. 2022 Sep 2;13:894325. doi: 10.3389/fgene.2022.894325 (PMC9478798; doi:10.3389/fgene.2022.894325)

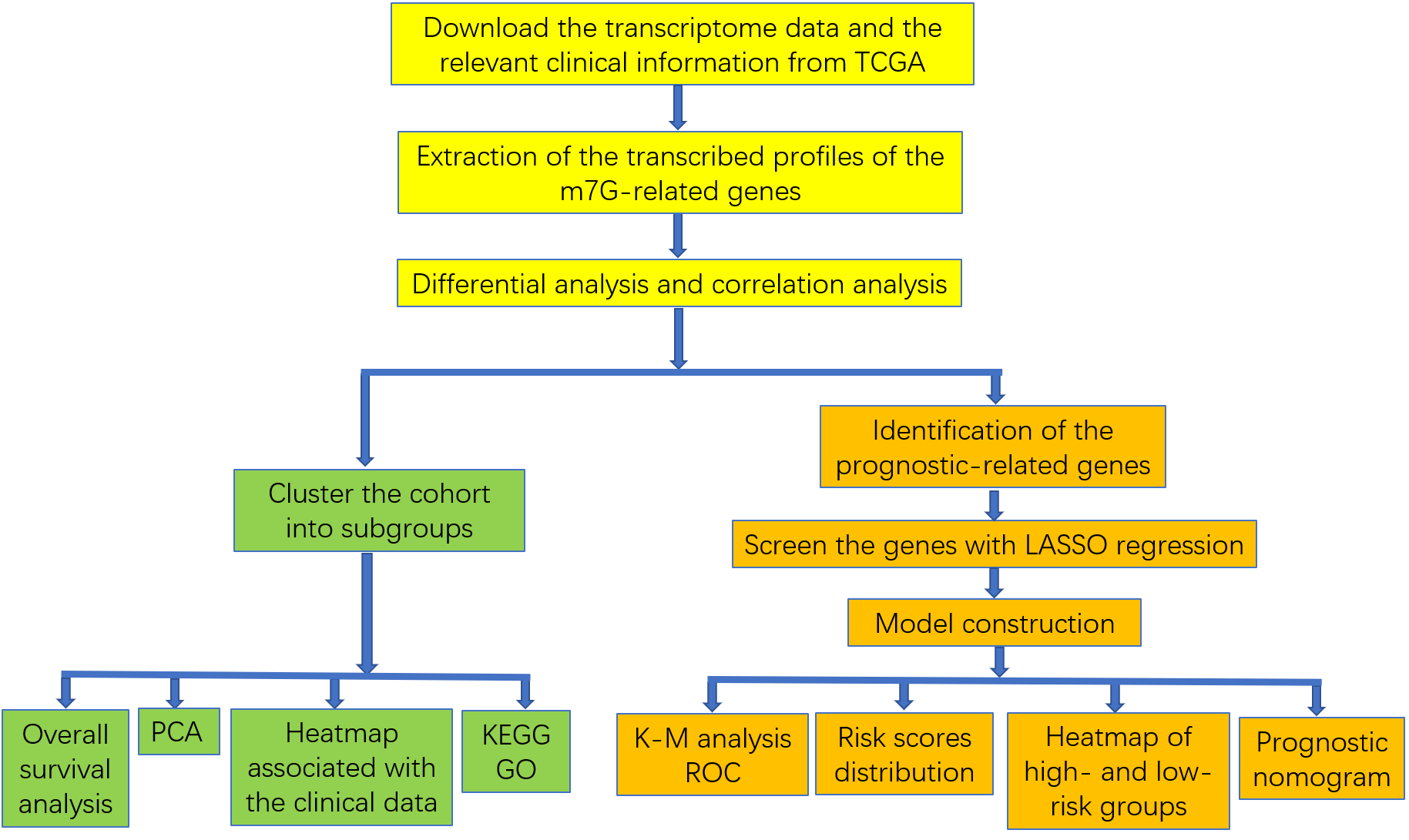

Supplement: Supplementary file 1 [file Image1.TIF]
